# Antihypertensive medications and risk of death and hospitalizations in US hemodialysis patients: Evidence from a cohort study to inform hypertension treatment practices

**DOI:** 10.1097/MD.0000000000005924

**Published:** 2017-02-03

**Authors:** Tariq Shafi, Stephen M. Sozio, Jason Luly, Karen J. Bandeen-Roche, Wendy L. St. Peter, Patti L. Ephraim, Aidan McDermott, Charles A. Herzog, Deidra C. Crews, Julia J. Scialla, Navdeep Tangri, Dana C. Miskulin, Wieneke M. Michels, Bernard G. Jaar, Philip G. Zager, Klemens B. Meyer, Albert W. Wu, L. Ebony Boulware

**Affiliations:** a Division of Nephrology, Johns Hopkins University School of Medicine; b Welch Center for Prevention, Epidemiology, and Clinical Research; c Department of Health Policy and Management; d Department of Biostatistics, Johns Hopkins Bloomberg School of Public Health, Baltimore, MD; e College of Pharmacy, University of Minnesota; f Chronic Disease Research Group, Minneapolis Medical Research Foundation, Minneapolis, MN; g Department of Epidemiology, Johns Hopkins Bloomberg School of Public Health, Baltimore, MD; h Department of Internal Medicine, Hennepin County Medical Center, University of Minnesota; i Cardiovascular Special Studies Center, United States Renal Data System, Minneapolis, MN; j Department of Nephrology, Duke University School of Medicine, Durham, NC; k Department of Medicine, Division of Nephrology, Seven Oaks General Hospital, University of Manitoba, Winnipeg, Manitoba, Canada; l Division of Nephrology, Tufts University School of Medicine, Boston, MA; m Division of Nephrology, Department of Medicine, Academic Medical Center, Amsterdam, The Netherlands; n Nephrology Center of Maryland, Baltimore, MD; o Division of Nephrology, University of New Mexico, Albuquerque, New Mexico; p Department of Health Policy and Management; q Department of International Health; r Department of Surgery, Johns Hopkins University School of Medicine, Baltimore, MD; s Division of General Internal Medicine, Duke University School of Medicine, Durham, NC, USA.

**Keywords:** angiotensin converting enzyme inhibitors, angiotensin receptor blockers, antihypertensives, β-blockers, epidemiology and outcomes, hemodialysis, hypertension

## Abstract

Antihypertensive medications are commonly prescribed to hemodialysis patients but the optimal regimens to prevent morbidity and mortality are unknown. The goal of our study was to compare the association of routinely prescribed antihypertensive regimens with outcomes in US hemodialysis patients.

We used 2 datasets for our analysis. Our primary cohort (US Renal Data System [USRDS]) included adult patients initiating in-center hemodialysis from July 1, 2006 to June 30, 2008 (n = 33,005) with follow-up through December 31, 2009. Our secondary cohort included adult patients from Dialysis Clinic, Inc. (DCI), a national not-for-profit dialysis provider, initiating in-center hemodialysis from January 1, 2003 to June 30, 2008 (n = 11,291) with follow-up through December 31, 2008. We linked the USRDS cohort with Medicare part D prescriptions-fill data and the DCI cohort with USRDS data. Unique aspect of USRDS cohort was pharmacy prescription-fill data and for DCI cohort was detailed clinical data, including blood pressure, weight, and ultrafiltration. We classified prescribed antihypertensives into the following mutually exclusive regimens: β-blockers, renin–angiotensin system blocking drugs-containing regimens without a β-blocker (RAS), β-blocker + RAS, and others. We used marginal structural models accounting for time-updated comorbidities to quantify each regimen's association with mortality (both cohorts) and cardiovascular hospitalization (DCI-Medicare Subcohort).

In the USRDS and DCI cohorts there were 9655 (29%) and 3200 (28%) deaths, respectively. In both cohorts, RAS compared to β-blockers regimens were associated with lower risk of death; (hazard ratio [HR]) (95% confidence interval [CI]) for all-cause mortality, (0.90 [0.82–0.97] in USRDS and 0.87 [0.76–0.98] in DCI) and cardiovascular mortality (0.84 [0.75–0.95] in USRDS and 0.88 [0.71–1.07] in DCI). There was no association between antihypertensive regimens and the risk of cardiovascular hospitalizations.

In hemodialysis patients undergoing routine care, renin–angiotensin system blocking drugs-containing regimens were associated with a lower risk of death compared with β-blockers-containing regimens but there was no association with cardiovascular hospitalizations. Pragmatic clinical trials are needed to specifically examine the effectiveness of these commonly used antihypertensive regimens in dialysis patients.

## Abstract 2

Supplemental Digital Content is available in the text

## 1 Introduction

Hypertension is present in over 90% of dialysis patients and results in substantial morbidity.^[1–3]^ Treatment of hypertension in dialysis patients is complex, characterized by substantial heterogeneity in clinical practice patterns, which are fueled by a lack of definitive scientific evidence to guide care.^[4]^ Prescribers’ choices of antihypertensive regimens for hemodialysis patients may be driven by several factors, including comorbidities, cardiovascular disease (CVD),^[5]^ multidrug medication regimens,^[6]^ frequent transitions of care,^[7,8]^ as well as perturbations in multiple domains, including biochemical (eg, hyperkalemia), physiologic (eg, intradialytic hypotension,^[9]^ blood pressure [BP] variability,^[10]^ and myocardial stunning^[11]^), physical (eg, cramping, postdialysis fatigue,^[12]^ and cognitive^[13]^), and psychological (eg, depression,^[14]^ lack of self-efficacy^[15]^). Citing a lack of definitive evidence to guide clinical practice, the Kidney Disease: Improving Global Outcomes board declined to review management of hypertension in dialysis patients,^[16]^ calling attention to the need for increased focus to establish an improved evidence base for care.

Classic “explanatory” clinical trials establishing the efficacy of single drug regimens suggest that β-blockers are efficacious in improving cardiovascular outcomes in dialysis patients with cardiomyopathy.^[17–19]^ In contrast, clinical trials conducted in the general population have consistently demonstrated the efficacy of renin–angiotensin system blocking drugs on reducing cardiovascular outcomes.^[20–23]^ Our recent national analysis identified considerable variation and complexity in providers’ prescribed antihypertensive regimens for hemodialysis patients, with over 40 distinct combinations of different antihypertensives prescribed and a high rate (>30%) of antihypertensives class switches for individual patients.^[6]^ Ideally, pragmatic clinical trials, designed to identify the most effective treatment strategies as might be employed in the “real-world”, would be conducted to identify optimal hypertension management.^[24,25]^ However, given the expense and infrastructure required for pragmatic trials, preliminary evidence is needed about the association of common practices with important clinical outcomes. Substantial variation in current practice provides an opportunity to evaluate these alternative antihypertensive regimens.

We conducted an observational study in 2 national cohorts of hemodialysis patients to quantify associations between commonly prescribed β-blocker and renin–angiotensin system blocking drugs containing antihypertensive regimens with patients’ morbidity and mortality. We hypothesized, based on general population data, that renin–angiotensin system blocking drugs containing antihypertensive regimens would be associated with lower risk of death (all-cause and cardiovascular) and cardiovascular hospitalizations in hemodialysis patients.

## 2 Methods

### 2.1 Study design and population

Our primary cohort, constructed by linking data from the US Renal Data System (USRDS) with Medicare Part D data, included adult patients initiating in-center hemodialysis from July 1, 2006 to June 30, 2008 (Table S1). Our secondary cohort, constructed by linking electronic medical records (EMR) data with USRDS data, included adult patients initiating in-center hemodialysis from January 1, 2003 to June 30, 2008 in facilities operated by Dialysis Clinic, Inc. (DCI) a medium-sized, not-for-profit, and national dialysis provider.^[26]^ For both cohorts, we used USRDS claims data for comorbidities and hospitalizations, and the National Death Index, the “gold standard” measure of US mortality causes,^[27,28]^ to assess the cause of death.

A unique aspect of the USRDS cohort was that it reflected antihypertensives prescription-fill claims through Medicare Part D, representing providers’ prescription patterns and patients’ adherence patterns.^[29,30]^ The DCI cohort unique aspects included antihypertensives as documented in the EMR and clinical data which confound the association between antihypertensives and outcomes (such as BP, dry weight, volume removal, and other laboratory data), which the USRDS registry data did not provide.

The Johns Hopkins Medicine Institutional Review Board reviewed and approved the study.

### 2.2 Discrete time dataset construction

**Figure 1:**
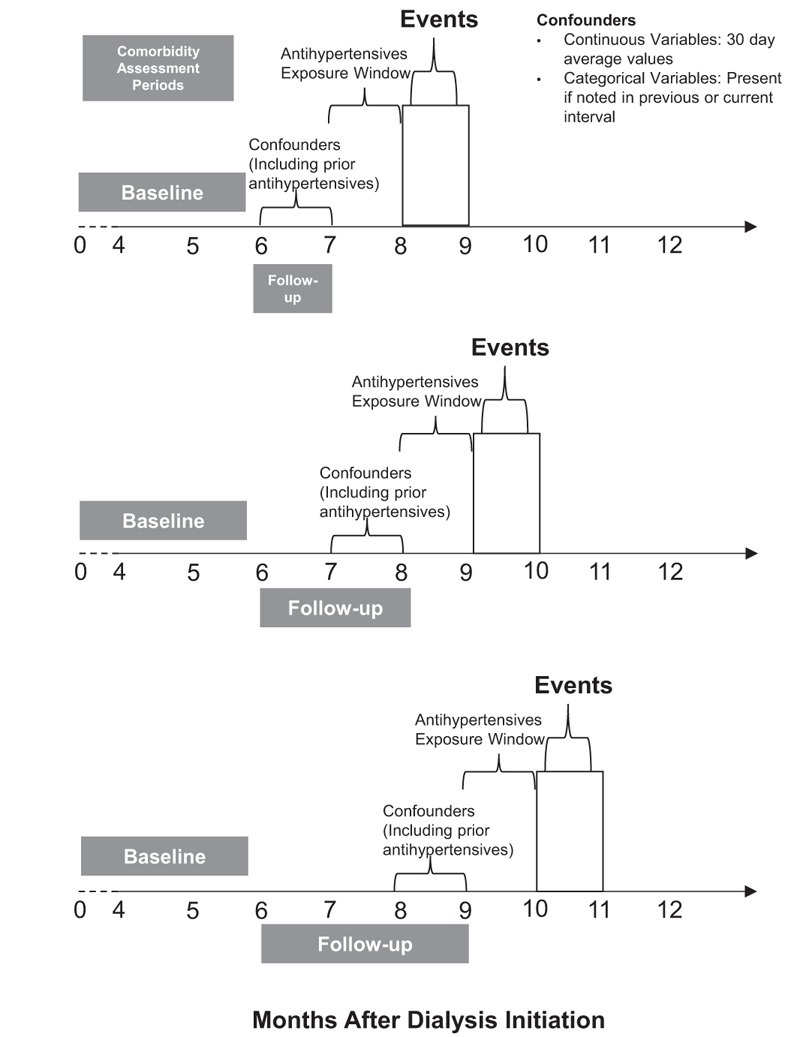
Timing of Assessment of Exposures and Outcome. Horizontal axis represents months after initiation of dialysis. The gray bars represent comorbidity assessment periods. Antihypertensive exposure window refers to the 30-day interval in which the antihypertensive regimen is assessed. Predictors used to determine to propensity (probability) of antihypertensive regimen prescription are always assessed in the periods prior to the antihypertensive exposure window.

We defined the baseline comorbidity assessment period as consisting of patients’ 1st 180 days after starting hemodialysis (Fig. 1). Starting from day 181 after patients initiated hemodialysis, we followed patients in both cohorts for outcomes until the end of available follow-up data – December 31, 2009 for the USRDS cohort and December 31, 2008 for the DCI cohort. For both cohorts, we censored patients if they underwent kidney transplantation, switched to home dialysis, were lost to follow-up, or, for the DCI cohort, if they were transferred to a non-DCI facility. We divided patients’ follow-up time into 30-day discrete time intervals. During each 30-day interval, we updated patients’ comorbidities and antihypertensives, leading up to and preceding the outcome interval.

### 2.3 Comorbidity assessment

A core consideration of our analysis was assessing comorbidity that could influence providers’ antihypertensive prescribing practices, which are particularly dynamic over the 1st 6 months of treatment. During this time, morbidity and mortality can be influenced by multiple factors (eg, predialysis care, dialysis access complications) that are unrelated to the biological effects of antihypertensives. However, these factors can influence prescribers’ choice of antihypertensive regimens for the patients. Additionally, it is well recognized that assessing comorbidity data at dialysis initiation solely from CMS Form-2728 can significantly underestimate patients’ morbidities.^[31,32]^

Therefore, in an attempt to accurately characterize baseline comorbidity and reduce confounding, we defined the baseline comorbidity assessment period as comprising patients’ 1st 180 days after starting hemodialysis (Fig. 1). During this period, we identified comorbidities using: data from Form-2728, supplemented by; Medicare hospitalization claims (both cohorts); and hospitalization data from DCI EMR (DCI cohort). All patients included in our analyses were therefore alive on day 180 (6 months) after initiating hemodialysis. During each subsequent 30-day follow-up interval, starting at day 181, we updated the presence or incidence of comorbidities using EMR and claims (including the presence or development of diabetes, CVD, congestive heart failure [CHF], chronic obstructive pulmonary disease).

### 2.4 Exposure: antihypertensive medication regimens

In determining a classification scheme for antihypertensives, we considered the proposed unique vascular effects of various classes of antihypertensives. For instance, β-blockers have beneficial effects in patients with coronary artery disease, while renin–angiotensin system blocking agents have effects on cardiac remodeling and reduce risk of cardiovascular outcomes.^[20,22,23]^ We hypothesized that providers might distinguish these unique effects when prescribing regimens, above and beyond their antihypertensive effects, while simultaneously balancing potential toxicity of these drugs. Similar choices may not play a role in the prescription of calcium channel blockers. Our prior work demonstrates that as many as 50% of all dialysis patients receive calcium channel blockers^[6]^ making it difficult to further subcategorize antihypertensive regimens.

We therefore classified antihypertensives into the following mutually exclusive regimens: β-blocker containing regimens without a RAS drug (BB), renin–angiotensin system blocking drugs containing regimens without a β-blocker (RAS), both β-blocker and renin–angiotensin system blocking drugs-containing regimens (BB + RAS), and other antihypertensive regimens without β-blocker or renin–angiotensin system blocking drugs (OTHER). We defined patients’ baseline antihypertensive regimen as the regimen recorded on day 180. We categorized patients that discontinued antihypertensives during follow-up as a discontinued medications group (DC). We updated patients’ regimens during each 30-day follow-up interval up to and preceding the interval in which the outcome occurred (Fig. 1).

For the USRDS cohort, we extracted antihypertensive prescriptions filled by patients from Medicare Part D data. For the DCI cohort, we assessed prescriptions from nurse-entered EMR data. In a subset of DCI patients with Medicare Part D we noted high concordance in medications between the EMR and Medicare Part D; 90% for β-blockers and 86% for RAS drugs.

### 2.5 Outcomes

Our primary outcomes in both cohorts were all-cause and cardiovascular death (defined as primary cause of death from heart disease, peripheral vascular disease, or cerebrovascular disease; Table S2).^[26]^

Our secondary outcome was a composite endpoint of cardiovascular hospitalization (identified using Medicare claims [Table S2] and DCI EMR)^[26]^ or all-cause death. For this outcome, we limited our analysis to DCI cohort with Medicare A and B coverage (DCI-Medicare) as the detailed dialysis treatment level data, including adherence, BP, and volume changes, allowed us to carefully account for comorbidity preceding hospitalizations.

### 2.6 Other covariates

We prespecified covariates (Table S3) to be included in outcome models based on clinical evidence that they may act as confounders or mediators. For continuous variables, we used average values during each 30-day interval. For categorical variables, we considered them as present if they were present at baseline or leading up to and including the time interval under consideration. Importantly, for our DCI cohort, covariates included comorbidities including CVD and hospitalizations, detailed dialysis session data including treatment adherence, predialysis systolic BP, dry weight attainment, and ultrafiltration as well as laboratory data including serum albumin, hemoglobin, Kt/V_UREA_, and calcium–phosphate product (Table S3).

### 2.7 Statistical analysis

Although the DCI cohort was included in the administrative USRDS national cohort, there were different data available to inform the analyses of the 2 cohorts. Thus, we conducted analyses in parallel in the 2 cohorts and did not combine the results. We described patients’ baseline characteristics by antihypertensive regimens.

**Figure 2:**
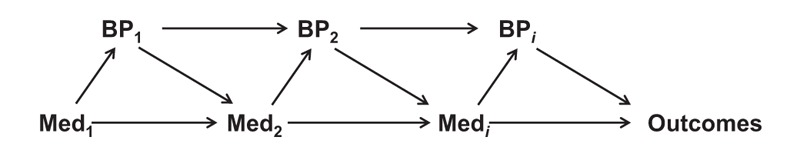
Simplified DAG of the Time-Varying Association Between Antihypertensive Regimens, BP, and outcomes. In this simplified model, the association of antihypertensive regimen (Med) at time1 influences the BP at time1. Both Med1 and BP1 influence the Med and BP at time2, and so on. This complex interplay finally contributes to the observed outcomes. BP = blood pressure, DAG = Directed Acyclic Graph.

We hypothesized that several time-varying factors, such as BP and volume status that are associated with outcomes, are likely to influence prescribers’ antihypertensive regimens choice (confounders) but could also mediate the effect of antihypertensives on outcomes (mediators; Fig. 2). In the presence of time-varying confounding and mediation, traditional multivariable adjustment may not well-approximate a randomized inference.^[33]^ We therefore used marginal structural models to quantify the association between antihypertensive regimens and outcomes. Marginal structural models’ analyses account for observed time-varying confounding and are designed to produce unbiased estimators of the causal mortality rate ratio across treatments (ie, per treatment pairing, a ratio comparing a population's mortality rate when all its members receive a given treatment to the rate when all its members receive another given treatment). The analysis envisions a study in which individuals are successively randomized to treatment categories in each month, and it estimates, say, the next-monthly relative mortality risk between treatment groups under these circumstances. The causal interpretation of the hazard ratio from these models is the ratio of the outcome rate had all members of the population represented by our subjects been continuously exposed compared to the outcome rate if all remained unexposed.^[34]^ As elucidated by Hernan and coworkers,^[35]^ the estimators do indeed converge to the causality mortality rate ratio when the outcomes and probabilities of treatment taken are correctly modeled in terms of the available covariates and there is no unmeasured confounding. We rigorously diagnosed the fit of our models for both the probabilities of treatment taken and outcomes and iterated to achieve improved fit, using interactions and flexible functions to capture nonlinearity where needed. Therefore, we believe that we achieved a reasonable approximation to the model fit assumptions. The assumption of no unmeasured confounding cannot be empirically verified: it challenges any statistical analysis that might be applied to our data. Our analysis likely is most at risk with respect to provider judgements in matching treatments to patients’ status, which are difficult to capture empirically.

For each 30-day interval, we used multinomial logistic regression to determine an individual's probability (propensity) for receiving a particular antihypertensive regimen as a function of covariates including past month's antihypertensive regimen. We then used this propensity to calculate stabilized inverse probability weights (see Supplemental Methods for details). We used discrete time proportional hazards models incorporating these weights to determine the association between antihypertensive regimens and outcomes. We conducted analyses on hospitalization only in the DCI cohort as detailed BP and treatment level data preceding hospitalization is not available for the USRDS cohort. For hospitalization analyses, models were constructed similarly. We accounted for recurrent hospitalizations within individuals, using a modified version of the Andersen–Gill approach.^[36]^ We prespecified subgroup analyses based on age, sex, race-ethnicity, diabetes, CVD, and CHF. In sensitivity analyses, we examined unweighted associations and associations after truncating for extreme weights (>99th percentile).

We performed all statistical analyses using SAS 9.2 (SAS Institute Inc., Cary, NC). We defined statistical significance as *P* < 0.05 using 2-tailed tests.

## 3 Results

### 3.1 Baseline characteristics

**Figure 3:**
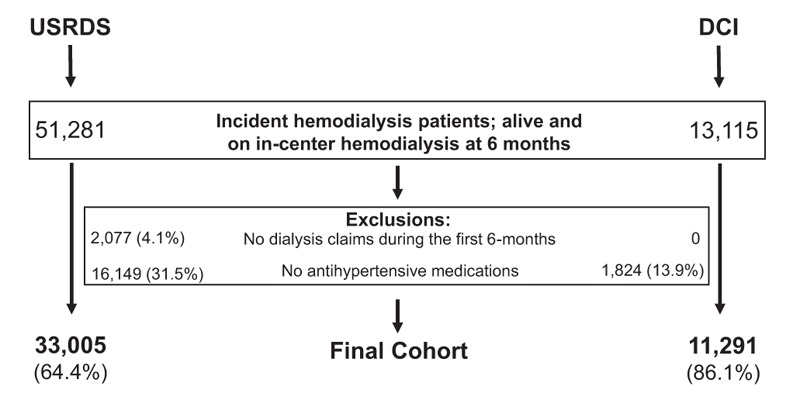
Selection of the final USRDS and DCI cohorts. DCI = Dialysis Clinic, Inc., USRDS = United States Renal Data System.

The final study populations included 33,005 (USRDS) and 11,291 (DCI) patients who were alive and receiving in-center hemodialysis at day 180 after dialysis initiation (Fig. 3). Most patients were receiving β-blocker containing regimens (either BB or BB + RAS) at baseline (day 180; Table 1). Patients on β-blocker regimens tended to be older, and had more CVD and CHF, and higher comorbidity index scores. Patients with diabetes were more likely to be on RAS containing regimens (either RAS or BB + RAS). At baseline, patients were receiving 88 (both USRDS and DCI) unique antihypertensives, and numerous unique antihypertensive medication combinations (USRDS, 5944; DCI, 3760) that reflected numerous antihypertensive medication class combinations (USRDS, 225; DCI, 188).

**Table 1:**
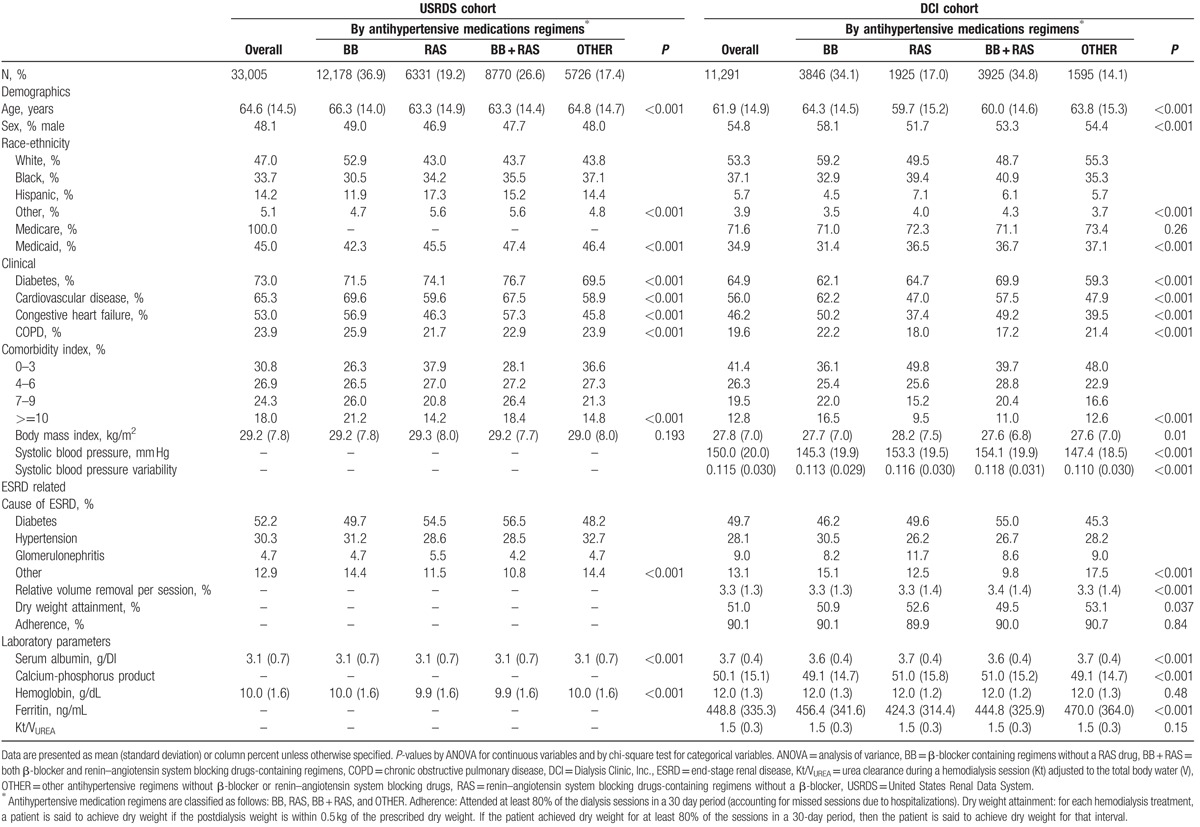
Baseline characteristics of the patients in the USRDS and DCI cohorts.

Our strategy to supplement form 2728 data with comorbidities claims in the baseline period significantly increased the assessment of comorbidities (*P* < 0.001) including CVD (absolute increase in prevalence, USRDS 28%; DCI 17%), CHF (absolute increase in prevalence, USRDS 18%; DCI 12%), and diabetes (absolute increase in prevalence, USRDS 13%; DCI 7%).

### 3.2 All-cause mortality

**Figure 4:**
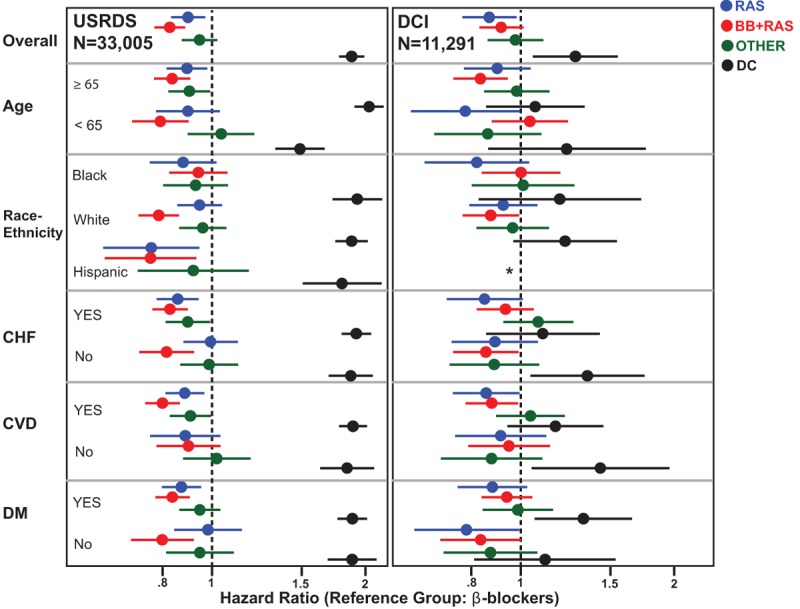
Association of Antihypertensive Regimens with All-Cause Mortality in U.S. Incident Hemodialysis Patients. Overall and subgroup analyses of the risk of all-cause mortality with antihypertensive regimens. Results from the USRDS cohort are displayed in the left panel and the DCI cohort in the right panel. Dots represent point estimates of hazard ratio and bars represent 95% confidence interval. Reference group for all comparisons is: β-blocker containing regimens (BB) without a renin–angiotensin system blocking drug. Blue color represents RAS containing regimens without a β-blocker (RAS), red color represents both β-blocker and RAS containing (BB + RAS) regimens, green color represents OTHER, and black color represents group with discontinued antihypertensives during follow-up (DC). Note: In the DCI subgroup analysis, there were too few individuals to compute the associations in the Hispanic subgroup. This is indicated by ∗ in the figure. CHF = congestive heart failure, CVD = cardiovascular disease, DCI = Dialysis Clinic, Inc., DM = diabetes mellitus, USRDS = United States Renal Data System.

All-cause mortality rates were similar in the 2 cohorts. During follow-up, there were 9655 (29.5%) deaths in the USRDS cohort and 3200 (28.3%) deaths in the DCI cohort. Compared to BB regimens, RAS regimens were associated with 10% and 13% lower risk of death in the USRDS and DCI cohorts, respectively, while BB + RAS regimens were associated with a 17% and 8% lower risk of death in the USRDS and DCI cohorts, respectively (Fig. 4; Tables 2 and 3). Prescription of OTHER regimens was not associated with differential risk of death compared to prescription of BB regimens, in fully adjusted multivariable models incorporating time-updated covariates. Subgroup analyses in both cohorts (Fig. 4; Table S5) showed similar direction of associations. Of note, the DC was associated with higher risk of death in the USRDS cohort but in the DCI cohort, with accounting for treatment level time-updated covariates, the risk association was significantly attenuated.

**Table 2:**
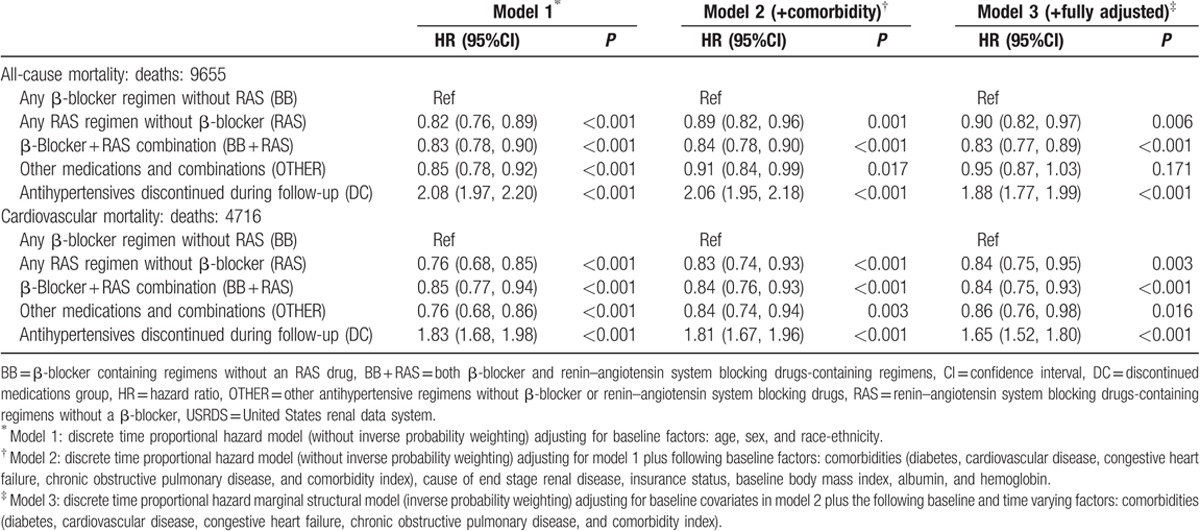
Association of antihypertensive medication regimens with all-cause and cardiovascular mortality among incident hemodialysis patients of the USRDS cohort (N = 33,005).

**Table 3:**
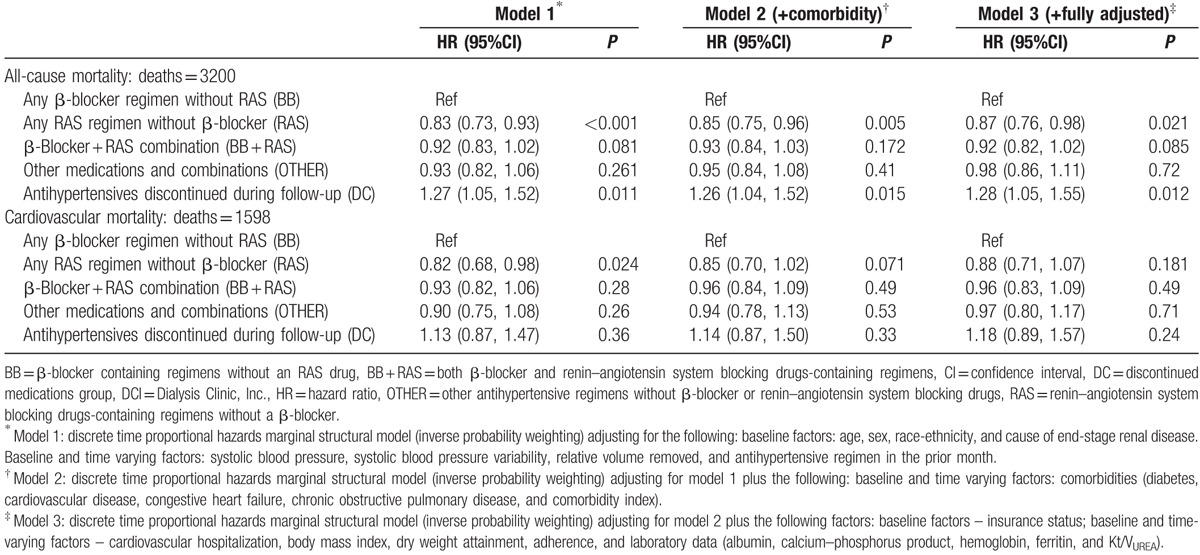
Association of antihypertensive medication regimens with all-cause and cardiovascular mortality among 11,291 incident hemodialysis patients of the DCI cohort.

### 3.3 Cardiovascular mortality

Cardiovascular death rates were also similar in the 2 cohorts. There were 4716 deaths (48.8% of all deaths) due to cardiovascular causes in USRDS and 1598 deaths (49.9% of all deaths) due to cardiovascular causes in DCI. In the USRDS cohort, RAS, RAS + BB, and OTHER regimens were associated with a 16%, 16%, and 14% lower risk of cardiovascular mortality, respectively, compared with BB regimens (Table 2). In DCI, direction of association was similar but did not reach statistical significance (Table 3). Subgroup analyses in both cohorts (Table S6) showed generally similar direction of association.

### 3.4 Cardiovascular hospitalizations or death (DCI-medicare cohort only)

Among 7848 patients in the DCI-Medicare subcohort, there were 15,158 events which included up to 4 repeat cardiovascular hospitalizations per patient and 1672 deaths (Table S7). In the final model, the risk of hospitalization was not significantly lower with RAS regimens compared to BB regimens, overall, or in subgroups. However, there were trends toward lower risk of hospitalization with RAS regimens compared to BB regimens among Blacks, and among those with CVD or diabetes at baseline.

### 3.5 Sensitivity analyses

The results were unchanged in unweighted models, after truncation of inverse probability weights, and after restricting the mortality analysis of the DCI cohort to only those patients with Medicare claims (data not presented).

## 4 Discussion

In this comprehensive national study of US in-center hemodialysis patients, we found that patients prescribed RAS regimens had a lower risk of all-cause and cardiovascular mortality but equivalent cardiovascular hospitalizations, when compared to patients prescribed BB regimens. Our findings were robustly consistent in full USRDS analyses incorporating only claims data, and in more detailed analyses incorporating both claims and detailed treatment-level clinical variables in DCI.

As a comparative effectiveness study representing real-world medication use in 2 separate cohorts, our study suggests that renin–angiotensin system blocking agents may be preferred antihypertensives in hemodialysis patients. However, our study also contributes to the mixed evidence on the effectiveness of antihypertensives among hemodialysis patients.^[16]^ In prior trials among hemodialysis patients, the β-blocker carvedilol and angiotensin receptor antagonist telmisartan were demonstrated to be beneficial in patients with cardiomyopathy.^[37,38]^ However, the angiotensin-converting enzyme inhibitor fosinopril did not reduce cardiovascular outcomes in patients with left ventricular hypertrophy.^[39]^ More recently, a randomized trial comparing β-blocker atenolol and angiotensin converting enzyme inhibitor lisinopril in hemodialysis patients was stopped early due to higher risk of the composite cardiovascular outcome in those treated with lisinopril.^[19]^ These findings of the lack of beneficial effects of renin–angiotensin system blocking drugs in dialysis patients are contradictory to numerous large clinical trials of antihypertensives in the general population,^[20,23]^ adding considerable uncertainty to clinical practice.^[4]^

Larger scale clinical trials among dialysis patients are needed to clarify uncertainty in clinical management of hypertension.^[40]^ However, given the expense and time required to conduct large clinical trials, findings from rigorous observational analyses such as ours, which attempted to model the complexity of real-world treatment circumstances, may provide important insights to inform future trials. For instance, our choice of comparator antihypertensive treatment groups was driven not only by prior evidence of potential effectiveness of both β-blocker and renin–angiotensin system blocking drugs in CVD among the general population^[20,22,23]^ and dialysis patients,^[17,18,41]^ but was also driven by the frequency with which we observed these combinations in practice.^[6]^ We intentionally considered the dynamic interplay of frequent changes in treatment regimens,^[6]^ changing comorbidities, and significant variability^[10]^ in key physiological variables (eg, BP and dry weight) in our analyses. If future trials are to definitively corroborate or refute our findings and inform clinical practice, they will need to capture these influences on treatment strategies and outcomes. Excluding such patients in future trials will render their results relatively meaningless for the majority of hemodialysis patients.^[25]^

There are potential biological explanations for our findings. β-Blockers and renin–angiotensin system blocking drugs may have differential benefits beyond their BP lowering effects.^[42,43]^ Renin–angiotensin system blocking drugs confer effects on left ventricular remodeling after myocardial infarction and other vascular effects^[44,45]^ that could influence outcomes. In the general population, β-blockers have been implicated in worsening diabetes control and greater insulin resistance when compared to other antihypertensives.^[46–48]^ These effects may be more pronounced among dialysis patients who have a very high prevalence of diabetes and suffer higher rates of CVD. Lack of benefit of β-blockers compared to other antihypertensives in our study could also be explained by differential effects of antihypertensives in the setting of altered calcification and vascular biology that occurs in patients on hemodialysis.^[49]^

Our overall approach utilized important differences in USRDS and DCI data to bolster our findings. For instance, claims data on antihypertensives in USRDS reflect prescription fill rates more closely than medications obtained from DCI medical records (which may less accurately reflect patients’ actual medication use than claims). In contrast, DCI data accounted for changes in comorbidities, BP, volume status (including dry weight attainment and volume removed), adherence with dialysis, and prior antihypertensive use that could not be accounted for with claims. Although these analyses corroborated one another, we cannot eliminate the concern that our observational study design may not have fully addressed confounding by indication as it relates to the use of β-blockers.^[50–53]^ Specifically, providers may have been driven in their prescribing by their judgment as to agents’ unique physiological effects and patient characteristics, based on criteria not well represented in our data.

Additional limitations of our study warrant consideration. First, we restricted our population to patients surviving for at least 180 days, limiting the generalizability of our findings to hemodialysis patients who have survived to 6 months. We deliberately chose this approach to better account for important comorbidities that could heavily influence clinicians’ antihypertensive prescription decisions. Second, as we used data from dialysis clinical practice, data collection was not standardized and cardiovascular outcomes were not adjudicated. We recognize that BP measures obtained at the time of dialysis may not reflect nondialysis BPs.^[1,2,54]^ However, nephrologists base their prescribing decisions on dialysis unit BP values. Third, our approach, while improving comorbidity assessment, precludes assessment of antihypertensive regimens in the early period after dialysis initiation and other analyses such as the impact of early versus later start of renin–angiotensin system blocking drugs on outcomes. Fourth, RAS regimens may increase serum potassium but we were not able to assess this change as the USRDS cohort did not have follow-up laboratory data, and both cohorts did not have data on 2 important determinants of hyperkalemia in dialysis patients, dietary intake, and residual kidney function. These limitations are balanced by our meticulous analytic approach with comprehensive inclusion of multiple patient characteristics and biological measures, the use of highly rigorous, prespecified analytic methods, large sample size, and parallel analyses in 2 cohorts to allow replication of findings and improve generalizability to real-world clinical settings.

In conclusion, we found that renin–angiotensin system blocking drugs-containing regimens, prescribed in routine clinical practice to hemodialysis patients, were associated with lower risk of death, compared to β-blocker-containing regimens. However, we found no difference in cardiovascular hospitalizations between antihypertensive regimens. Our findings support the conduct of carefully designed pragmatic clinical trials that account for considerable complexity in the real-world treatment of hypertension among these high-risk patients.

## Acknowledgments

The DEcIDE Network Patient Outcomes in End-Stage Renal Disease Study Team consists of members from the Johns Hopkins University, Baltimore (L. Ebony Boulware, Karen Bandeen-Roche, Courtney Cook, Josef Coresh, Deidra Crews, Patti Ephraim, Bernard Jaar, Jeonyong Kim, Yang Liu, Jason Luly, Aidan McDermott, Wieneke Michels, Paul Scheel, Tariq Shafi, Stephen Sozio, Albert W. Wu, Jing Zhou); University of California, San Francisco (Neil Powe); the Chronic Disease Research Group, Minneapolis (Allan Collins, Robert Foley, David Gilbertson, Haifeng Go, Joseph Grill, Charles Herzog, Jiannong Liu, Wendy St. Peter); Cleveland Clinic Foundation (Joseph Nally, Susana Arrigain, Stacey Jolly, Vicky Konig, Xiaobo Liu, Sankar Navaneethan, Jesse Schold); Dialysis Clinic, Incorporated, Nashville (Karen Majchrzak, Phil Zager); Tufts University, Boston (Dana Miskulin, Klemens Meyer); University of Miami (Julia Scialla); University of Manitoba (Navdeep Tangri); and Academic Medical Center, The Netherlands (Wieneke Michels).

The authors thank the staff and patients of Dialysis Clinic Inc.

## Supplementary Material

Supplemental Digital Content
