# Supplementary material for: Antihypertensive medications and risk of death and hospitalizations in US hemodialysis patients: Evidence from a cohort study to inform hypertension treatment practices
Source: Medicine (Baltimore). 2017 Feb 3;96(5):e5924. doi: 10.1097/MD.0000000000005924 (PMC5293434; doi:10.1097/MD.0000000000005924)
Supplement: Supplemental Digital Content [file medi-96-e5924-s001.docx]

**Antihypertensive Medications and Risk of Death and Hospitalizations in US Hemodialysis Patients: Evidence from a Cohort Study to Inform Hypertension Treatment Practices**

**SUPPLEMENTAL METHODS**

**Mortality Analyses in both the USRDS and DCI cohorts**

In a setting such as ours, marginal structural models are designed to produce unbiased estimates of the causal mortality rate ratio across treatments (that is, per treatment pairing, a ratio comparing a population’s mortality rate when all its members receive a given treatment to the rate when all its members receive another given treatment). Following common practice, we implemented this approach within discrete-time survival models taking person-months as observations. We employed discrete-time proportional hazards models as proposed by Prentice & Gloeckler^1^, modeling time as a quartic function of month; fits from these closely paralleled those of models employing time-dependent intercepts. Time-varying summaries of medications and predictors were created over 30-day intervals, which allowed smoothing of the covariates.

For marginal structural model analyses, a first step was to develop the treatment weights for antihypertensive medication regimens. As we categorized antihypertensive medication use to 5 medication regimens, our response in the treatment weight model was a multilevel factor. Thus, multinomial logistic regression was used to develop treatment predictions. We then constructed weights as per Hernan,^2^ with the denominator defined as the predicted probability (propensity) of being on one’s observed regimen as a function of past treatment and time-varying covariates, and the numerator defined as the predicted probability of being on one’s observed regimen as function of past treatment. We stabilized weights by including baseline covariates in models used to produce both numerator and denominator predictions. Analogous “censoring” weights were also developed, using a procedure identical to that just described except that logistic regression was used to develop predictions of the probability of censoring in an interval. The cumulative product of the treatment and censoring weights for each patient up to each exposure interval represented the final weights for the marginal structural model. Like propensity scoring, marginal structural model aims to “balance” covariates between treatment groups so as to mimic a randomized experiment, but it does so longitudinally so as to mimic re-randomization at every observation time.

Treatment weights were developed in nested sets in which we sequentially considered covariates to be associated with treatment. We selected factors to include in our treatment models *a priori* based on factors we believe to influence physicians’ antihypertensive medication prescribing practices. Our first set thus included those factors that likely primarily influence a prescriber’s choice of medication, including baseline demographic factors (age, sex, race, ethnicity, cause of ESRD), prior month’s antihypertensive medication regimen, and prior month’s blood pressure (BP) factors for the DCI cohort (systolic BP, BP variability and relative volume removed during each hemodialysis session). To this first set, our second set added prior month’s cumulative comorbidity [comorbidity index, congestive heart failure, cardiovascular disease, diabetes mellitus, chronic obstructive pulmonary disease (which may affect ability to prescribe β-blocker)]. Our third set of treatment weights added additional clinical characteristics including cardiovascular hospitalizations (for the DCI cohort), albumin, ferritin, hemoglobin, Kt/V, calcium–phosphorus product, dry weight attainment, adherence, insurance status, and body mass index.

Following construction of weights, we then fit discrete-time proportional hazards models (binomial regressions for event using complementary log-log link) incorporating these weights. We truncated weights at the 99^th^ percentile, with those extreme weights greater than the 99^th^ percentile assigned to the 99^th^ percentile. We performed checks for fit on the treatment model, the censoring model, and the discrete time proportional hazards model for outcomes.

For all discrete-time data sets, we used Markov chain Monte-Carlo Multiple Imputation^3^ to create 10-imputed datasets to fill in remaining missing variables. The percent of 30-day patient-intervals with missing data points were: systolic blood pressure, 0.4%; relative volume removed, 0.8%; body mass index, 1.0%; Kt/V, 0.2%; albumin, 8.4%; ferritin, 3.2%; hemoglobin, 3.7%; and calcium-phosphate product, 6.9%. The prediction of the imputation model was evaluated by sampling 100 non-missing observations for each variable subject to imputation, re-setting those observations as missing, running the imputation model, and evaluating the predicted against the actual observed values. In this substudy, no significant biases were observed however values of R^2^ ranged between 0.03 and 0.3. Thus our imputation accurately reproduced observed data means but left considerable variance unaccounted by observed information underlying it.

**Hospitalization Analyses in the DCI Cohort**

We used discrete-time proportional hazards models (binary regressions for event using complementary log-log link^4^) to model time to hospitalization, taking person-months between hospitalizations as observations (e.g. time between 181 days and the first hospitalization as first occurrence, time between first hospitalization and second hospitalization as first recurrence, and so on). To account for multiple hospitalizations, we applied a modified version of the Andersen-Gill approach.^5^ We allowed for changing baseline hazards depending on the hospitalization for which the patient was at risk by using hospitalization-specific intercepts (i.e., a term for 1^st^, 2^nd^, 3^rd^, etc, hospitalization) and time functions (modeled as a quartic function of month). As repeated hospitalization events may be correlated both within person and within clinic, we fit our repeated events models using Generalized Estimating Equations allowing for clustering within person and within clinic. To maintain reasonable power and avoid undue influence of small numbers of patients with high numbers of hospitalizations, we allowed each patient to have up to 4 hospitalizations, censoring after the 4^th^ hospitalization. We considered the possibility of hospitalization-specific effects of treatment, but a global test of interactions between hospitalization and treatment choice showed no evidence of a changing effect of treatment across hospitalization. Finally, we investigated the impact of more liberal weight truncation points on the model estimates and the effect of grouping hospitalizations after the 4th with the 4^th^ as opposed to censoring at the 4^th^ hospitalization.

**Model Checking**

We used generalized estimating equations^6^ in all analyses to account for outcome clustering by dialysis facilities and assure conservative inferences. We examined plots of Pearson residuals versus predicted values and Pearson residuals versus covariate values. We incorporated spline terms to accommodate nonlinear associations.

All statistical analyses were performed using SAS 9.2 (SAS Institute Inc., Cary, North Carolina). We defined statistical significance as *p*<0.05 using two-tailed tests.

**References**

**1.** Prentice RL, Gloeckler LA. Regression analysis of grouped survival data with application to breast cancer data. *Biometrics.* Mar 1978;34(1):57-67.

**2.** Hernan MA, Brumback B, Robins JM. Marginal structural models to estimate the causal effect of zidovudine on the survival of HIV-positive men. *Epidemiology.* Sep 2000;11(5):561-570.

**3.** Schafer JL. *Analysis of Incomplete Multivariate Data*. New York: Chapman and Hall; 1997.

**4.** Prentice RL, Kalbfleisch JD, Peterson AV, Jr., Flournoy N, Farewell VT, Breslow NE. The analysis of failure times in the presence of competing risks. *Biometrics.* Dec 1978;34(4):541-554.

**5.** Andersen PK, Gill RD. Cox's regression model for counting processes: a large sample study. *The annals of statistics.* 1982:1100-1120.

**6.** Zeger SL, Liang KY. Longitudinal data analysis for discrete and continuous outcomes. *Biometrics.* Mar 1986;42(1):121-130.

**Table S1: Data Setup and Design for the USRDS and DCI Cohorts**

|  | **USRDS** | **DCI** |
| --- | --- | --- |
| **Final Cohort N** | 33,005 | 11,291 |
| **Cohort Construction** |  |  |
| Ages included | >18 years | >18 years |
| Time Period | July 1, 2006 to June 30, 2008 | July 1, 2003 to June 30, 2008 |
| Insurance | Medicare Parts A, B, D | All Insurances |
| Linkage | National Death Index, Medicare Part D | USRDS, National Death Index, Medicare Part D (for patients starting dialysis after 2006) |
| **Baseline Comorbidity Assessment** | | |
| Assessment period | Day 1 to 180 after dialysis initiation | Day 1 to 180 after dialysis initiation |
| Data Source/s | CMS Form 2728, Medicare claims | CMS Form 2728, Medicare claims, DCI Electronic Medical Records (hospitalizations) |
|  |  |  |
| **Follow-up** |  |  |
| Start | Day 181 | Day 181 |
| End | Dec 31, 2009 or censor/outcome | Dec 31, 2008 or censor/outcome |
| Censor | Transplant, modality change, lost to follow up, end of study period (Dec 31, 2009) | Transplant, modality change, lost to follow up, transfer to non-DCI facility, end of study period (Dec 31, 2008) |
| **Exposure: Antihypertensive Medications** | | |
| Data Source/s | Pharmacy-fill information | Nurse entered data |
| Classification^1^ | Five regimens: BB, RAS, BB+RAS, OTHER, DC | Five regimens: BB, RAS, BB+RAS, OTHER, DC |
| **Outcome Definitions** |  |  |
| Outcome types | Mortality (all-cause and cardiovascular) | Mortality (all-cause and cardiovascular) and Cardiovascular hospitalizations |
| Data Source/s | National Death Index death certificate data | Mortality: National Death Index death certificate data  Hospitalizations: Medicare Claims Data and DCI EMR |
| **Analysis** |  |  |
| Data set-up | Discrete-time 30 day intervals starting from day 181 after start of hemodialysis | Discrete-time 30 day intervals starting from day 181 after start of hemodialysis |
| Time-updated covariates | Marginal structural models | Marginal structural models |
| Recurrent events | n/a | Anderson-Gill approach for repeat hospitalizations |
| Subgroups | Age, sex, race, baseline diabetes, cardiovascular disease and congestive heart failure | Age, sex, race, baseline diabetes, cardiovascular disease and congestive heart failure |

Abbreviations: USRDS, United States Renal Data System; DCI, Dialysis Clinic, Inc.; CMS, Centers for Medicare & Medicaid Services; EMR, Electronic Medical Record

^1^ Antihypertensive medication regimens are classified as follows: β-blocker containing (BB), renin-angiotensin system blocking drugs containing (RAS), β-blocker+ renin-angiotensin system blocking drugs containing (BB+RAS), other regimens (OTHER), or patients in whom medications were discontinued during follow-up (DC).

**Table S2: Codes for Assessing Cardiovascular Death and Cardiovascular Hospitalizations**

| **Category** | **Codes** |
| --- | --- |
| **Cardiovascular Death** | **ICD-10 Diagnosis Codes** |
| Chronic Rheumatic Heart Disease | I05-I09 |
| Hypertensive Heart Disease | I10, I11, I15 |
| Ischemic Heart Disease | I20-I25 |
| Other Heart Disease | I33-I37, I42-I51 |
| Cerebrovascular Disease | I60-I69 |
| Disease of Arteries, Arterioles and Capillaries | I70-I74, I77-I79 |
| Other and unspecified Circulatory Systems | I98-I99 |
| Vascular Disorders of Intestine | K55 |
| Peripheral vascular disease | R02 |
| No NDI match - Cardiac Death per USRDS |  |
|  |  |
| **Cardiovascular Hospitalizations** | **ICD-9 and CPT Codes** |
| Congestive Heart Failure or Cardiomyopathy | - ICD-9 primary codes: 398.91, 402.01, 402.11, 402.91, 404.01, 404.03, 404.11, 404.13, 404.91, 404.93, 425, 428, 518.4, 276.6 |
| Atherosclerotic Heart Disease | - ICD-9 primary codes: 410-414; - ICD-9 secondary codes: 410, 411.81; - CPT codes: 36.0, 36.9, 36.1, 36.2 |
| Conduction disorders and Dysrhythmias | - ICD-9 primary codes: 426-427, 798; - ICD-9 secondary codes: 427.4, 427.41-427.42, 427.5; - CPT codes: 37.6-37.8, 37.94-37.97, 00.50-00.54 |
| Cerebrovascular Disease | - ICD-9 primary codes: 430-438; - ICD-9 secondary codes: 430, 431, 432, 433.01, 434.01, 436, 437.2, 435–435.9; - CPT codes: 38.10, 38.11, 38.12 |
| Circulatory System | - ICD-9 primary codes: 440-456, 458–459, 250.7; - CPT codes: 38.13,38.18, 38.38,39.22, 39.25,39.25, 39.26,39.28, 39.29, 84.0, 84.1, 84.91 |
| Other Cardiac | - ICD-9 primary codes: 394-398, 415–420, 422–424, 429 |

Abbreviations: ICD, International Classification of Diseases; CPT, Current Procedural Terminology; NDI, National Death Index; USRDS, United States Renal Data Systems

**Table S3: Detailed Definition of Covariates and Timing of Assessment**

|  |  | **Baseline (BL) or Time Varying (TV)** | | | |
| --- | --- | --- | --- | --- | --- |
|  |  | **USRDS** | | **DCI** | |
| **Variables** | **Definition** | **BL** | **TV** | **BL** | **TV** |
| **Demographic** | | | | | |
| Age |  | **X** |  | **X** |  |
| Sex | Male or Female | **X** |  | **X** |  |
| Race-ethnicity | Non-Hispanic White  Non-Hispanic Black  Hispanic  Other | **X** |  | **X** |  |
| Insurance Status | Receiving Medicare or Medicaid benefits | **X** |  | **X** |  |
| **Clinical** | | | | | |
| Diabetes^1^ | Form 2728 + Medicare Claims + EMR for DCI cohort | **X** | **X** | **X** | **X** |
| Cardiovascular Disease^2^ | Form 2728 + Medicare Claims + EMR for DCI cohort | **X** | **X** | **X** | **X** |
| Congestive Heart Failure^3^ | Form 2728 + Medicare Claims + EMR for DCI cohort | **X** | **X** | **X** | **X** |
| Chronic Obstructive Pulmonary Disease^4^ | Form 2728 + Medicare Claims + EMR for DCI cohort | **X** | **X** | **X** | **X** |
| Cardiovascular Hospitalization^5^ | Medicare Claims + EMR for DCI cohort |  |  | **X** | **X** |
| Comorbidity^6^ | Liu Comorbidity Index | **X** | **X** | **X** | **X** |
| Body Mass Index | Weight in Kg/Height in m^2^ | **X** |  | **X** | **X** |
| Systolic Blood Pressure | Automated oscillometric blood pressure measured predialysis as part of clinical care |  |  | **X** | **X** |
| Systolic Blood Pressure Variability | Standard deviation of the residual from a linear mixed effect regression model of natural log transformed predialysis systolic blood pressure over time |  |  | **X** | **X** |
| **End-Stage Renal Disease Related** | | | | | |
| Cause of End-Stage Renal Disease | Diabetes, Hypertension, Glomerulonephritis, Other | **X** |  | **X** |  |
| Relative Volume Removed | (Predialysis Weight-Postdialysis Weight)/Postdialysis Weight |  |  | **X** | **X** |
| Dry Weight Attainment^7^ | Achieving dry weight in the previous 30 day interval |  |  | **X** | **X** |
| Adherence | Attended at least 80% of the dialysis sessions in a 30 day (accounting for missed sessions due to hospitalizations) |  |  | **X** | **X** |
| **Laboratory Data** | | | | | |
| Albumin | Serum Albumin (routine labs, measured monthly) | **X** |  | **X** | **X** |
| Calcium-Phosphate product | Corrected Serum Calcium x Serum Phosphate (routine labs, measured monthly) |  |  | **X** | **X** |
| Hemoglobin | Hemoglobin (routine labs, measured monthly) | **X** |  | **X** | **X** |
| Ferritin | Serum Ferritin (routine labs, measured every 3 months) |  |  | **X** | **X** |
| Kt/V_UREA_ | Calculated value from predialysis and postdialysis urea and other dialysis parameters. Values provided for use in clinical care were used. |  |  | **X** | **X** |
| **Antihypertensive Regimens** | | | | | |
| Antihypertensive Regimens | As described in Table S1 | **X** | **X** | **X** | **X** |

Abbreviations: USRDS, United States Renal Data System; DCI, Dialysis Clinic, Inc.; EMR, Electronic Medical Record; Kt/V_UREA_, Urea clearance during a hemodialysis session (Kt) adjusted to the total body water (V); ICD, International Classification of Diseases

^1^ Diabetes: Medicare claims with ICD-9 codes: 250; 357.2; 362.01-362.07; 366.41

^2^ Cardiovascular disease: Medicare claims for:

- Atherosclerotic heart disease (ICD-9 codes: 410–414, V45.81, V45.82)
- Cerebrovascular accident/transient ischemic attack (ICD-9 codes: 430–438)
- Peripheral vascular disease (ICD-9 codes: 440–444, 447, 451–453, 557)
- Dysrhythmia (ICD-9 codes: 426–427, V45.0, V53.3)
- Other cardiac disease (ICD-9 codes: 420–421, 423–424, 429, 785.0–785.3, V42.2, V43.3)

^3^ Congestive heart failure: Medicare claims with ICD-9 codes: 398.91; 422; 425; 428; 402.01; 402.11; 404.01; 404.11; 404.91; 404.03; 404.13; 404.93, V42.1

^4^ Chronic obstructive pulmonary disease: Medicare claims with ICD 9 codes: 491–494; 496; 510

^5^ Hospitalization: Medicare claims for:

- Congestive heart failure or cardiomyopathy (ICD-9 primary codes: 398.91, 402.01, 402.11, 402.91, 404.01, 404.03, 404.11, 404.13, 404.91, 404.93, 425, 428, 518.4, 276.6)
- Atherosclerotic heart disease (ICD-9 primary codes: 410-414; ICD-9 secondary codes: 410, 411.81; CPT codes: 36.0, 36.9, 36.1, 36.2)
- Conduction disorders and dysrhythmias (ICD-9 primary codes: 426-427, 798; ICD-9 secondary codes: 427.4, 427.41-427.42, 427.5; CPT codes: 37.6-37.8, 37.94-37.97, 00.50-00.54)
- Cerebrovascular disease (ICD-9 primary codes: 430-438; ICD-9 secondary codes: 430, 431, 432, 433.01, 434.01, 436, 437.2, 435–435.9; CPT codes: 38.10, 38.11, 38.12)
- Circulatory system (ICD-9 primary codes: 440-456, 458–459, 250.7; CPT codes: 38.13,38.18, 38.38,39.22, 39.25,39.25, 39.26,39.28, 39.29, 84.0, 84.1, 84.91)
- Other cardiac (ICD-9 primary codes: 394-398, 415–420, 422–424, 429)

^6^ Liu Comorbidity Index:[ Liu J, Huang Z, Gilbertson DT, Foley RN, Collins AJ. An improved comorbidity index for outcome analyses among dialysis patients. Kidney Int. 2010;77(2):141-51.]

- Assign score of 1 to atherosclerotic heart disease and diabetes;
- Assign score of 2 to cerebrovascular accident/transient ischemic attack, peripheral vascular disease, chronic obstructive pulmonary disease, gastrointestinal bleeding (ICD-9 codes: 456.0–456.2, 530.7, 531–534, 569.84, 569.85, 578), dysrhythmia, other cardiac, liver disease (ICD-9 codes: 570, 571, 572.1, 572.4, 573.1–573.3, V42.7) and cancer (ICD-9 codes: 140–172, 174–208, 230–231, 233–234);
- Assign score of 3 to congestive heart failure. A patients overall score is a sum of the individual scores.
- The overall score has range 0 to 21 with higher score representing greater comorbidity.

^7^ Dry weight attainment: For each hemodialysis treatment, a patient is said to achieve dry weight if the postdialysis weight is within 0.5 kg of the prescribed dry weight. If the patient achieved dry weight for at least 80% of the sessions in a 30-day period, then the patient is said to achieve dry weight for that interval.

**Table S4: Baseline Characteristics of 7,848 Incident Hemodialysis Patients of the DCI-Medicare Subcohort by Antihypertensive Regimen**

|  |  | **Overall** | **Antihypertensive Medication Regimen at 6 Months** | | | |  |
| --- | --- | --- | --- | --- | --- | --- | --- |
|  |  |  | Any β-blocker Regimen Without RAS (BB) | Any RAS Regimen Without β-blocker (RAS) | β-blocker +RAS Combination  (BB+RAS) | Other Medications and Combinations  (OTHER) | **p-value** |
|  |  |  |  |  |  |  |  |
| **N (%)** |  | 7848 | 2662 (33.9%) | 1338 (17.0) | 2704 (34.4) | 1144 (14.6) |  |
| **Demographics** |  |  |  |  |  |  |  |
| Age, years |  | 63.3 (15.0) | 65.8 (14.5) | 61.25 (15.4) | 61.1 (14.8) | 64.9 (15.3) | <0.001 |
| Sex, % male |  | 54.1 | 57.8 | 51.1 | 52.3 | 53.3 | <0.001 |
| Race-Ethnicity |  |  |  |  |  |  |  |
|  | Hispanic, % | 5.3 | 4.1 | 6.3 | 5.8 | 5.7 |  |
|  | White, % | 53.3 | 59.3 | 50.4 | 48.2 | 55.2 |  |
|  | Black, % | 37.6 | 33.3 | 39.6 | 42.0 | 35.2 |  |
|  | Other, % | 3.7 | 3.3 | 3.7 | 4.0 | 3.9 | <0.001 |
| Medicaid, % |  | 45.2 | 40.5 | 47.0 | 48.9 | 45.2 | <0.001 |
| **Clinical** |  |  |  |  |  |  |  |
| Diabetes, % | | 65.8 | 63.3 | 65.8 | 70.5 | 60.8 |  |
| Cardiovascular Disease, % | | 61.2 | 67.5 | 51.9 | 63.2 | 53.1 |  |
| Congestive Heart Failure, % | | 51.2 | 55.8 | 42.0 | 54.8 | 43.1 |  |
| Chronic Obstructive Pulmonary Disease, % | | 22.7 | 25.6 | 20.5 | 19.9 | 25.0 |  |
| Comorbidity Index, % | |  |  |  |  |  |  |
|  | 0-3 | 35.3 | 30.0 | 43.9 | 33.3 | 42.5 |  |
|  | 4-6 | 26.5 | 24.8 | 27.1 | 29.0 | 26.6 |  |
|  | 7-9 | 22.1 | 24.7 | 16.7 | 23.5 | 18.8 |  |
|  | >=10 | 16.1 | 20.5 | 12.3 | 14.2 | 15.1 | <0.001 |
| Body Mass Index, kg/m^2^ | | 27.6 (6.9) | 27.5 (6.9) | 28.0 (15.4) | 27.5 (6.7) | 27.4 (7.0) | 0.108 |
| Baseline Systolic Blood Pressure, mm Hg | | 150.0 (20.1) | 145.2 (19.8) | 153.4 (19.8) | 154.1 (19.9) | 147.7 (18.7) | <0.001 |
| Baseline Systolic Blood Pressure Variability | |  |  |  |  |  |  |
|  | Mean (SD) | 0.115 (0.03) | 0.113 (0.029) | 0.116 (0.030) | 0.119 (0.031) | 0.111 (0.030) | <0.001 |
|  | Median (25th-75th percentiles) | 0.112 (0.094, 0.134) | 0.110 (0.093, 0.131) | 0.113 (0.095, 0.134) | 0.116 (0.097, 0.138) | 0.106 (0.089, 0.127) | <0.001 |
| **ESRD-Related** | |  |  |  |  |  |  |
| Cause of ESRD, % | |  |  |  |  |  |  |
|  | Diabetes | 49.8 | 46.4 | 50.5 | 54.3 | 46.4 |  |
|  | Hypertension | 29.7 | 32.3 | 27.4 | 28.6 | 29.2 |  |
|  | Glomerulonephritis | 8.0 | 7.4 | 10.5 | 7.4 | 8.0 |  |
|  | Other | 12.4 | 13.9 | 11.5 | 9.8 | 16.4 | <0.001 |
| Relative Fluid Removal per Session, % | | 3.3 (1.4) | 3.3 (1.3) | 3.3 (1.4) | 3.4 (1.3) | 3.3 (1.4) | <0.001 |
| Dry Weight Attainment, % | | 51.6 | 52.2 | 51.9 | 50.1 | 53.2 | 0.25 |
| Adherence, % | | 92.4 | 92.8 | 91.7 | 92.5 | 92.4 | 0.70 |
| Relative Fluid Removal per Session, % | | 3.3 (1.4) | 3.3 (1.3) | 3.3 (1.4) | 3.4 (1.3) | 3.3 (1.4) | <0.001 |
| **Laboratory Parameters** | |  |  |  |  |  |  |
| Serum Albumin, g/dL | | 3.6 (0.4) | 3.6 (0.4) | 3.7 (0.4) | 3.6 (0.4) | 3.7 (0.4) | 0.004 |
| Calcium Phosphate Product | | 49.8 (14.9) | 48.9 (14.4) | 50.6 (15.6) | 50.7 (15.2) | 48.7 (14.6) | <0.001 |
| Hemoglobin, g/dL | | 12.0 (1.3) | 12.0 (1.3) | 12.1 (1.2) | 12.0 (1.3) | 12.0 (1.3) | 0.021 |
| Ferritin, ng/mL | | 453.2 (336.0) | 459.9 (343.3) | 423.8 (303.3) | 454.1 (334.3) | 470.0 (356.7) | 0.004 |
| Kt/V_UREA_ | | 1.5 (0.3) | 1.5 (0.3) | 1.5 (0.3) | 1.5 (0.3) | 1.5 (0.3) | 0.107 |

Abbreviations: USRDS, United States Renal Data System; DCI, Dialysis Clinic, Inc.; COPD, Chronic Obstructive Pulmonary Disease; Kt/V_UREA_, Urea clearance during a hemodialysis session (Kt) adjusted to the total body water (V)

Data are presented as mean (standard deviation) or column percent unless otherwise specified. P-values by ANOVA for continuous variables and by chi-square test for categorical variables.

^1^ Antihypertensive medication regimens are classified as follows: β-blocker containing (BB), renin-angiotensin system blocking drugs containing (RAS), β-blocker+ renin-angiotensin system blocking drugs containing (BB+RAS) and other regimens (OTHER).

Attended at least 80% of the dialysis sessions in a 30 day (accounting for missed sessions due to hospitalizations).

Dry weight attainment: For each hemodialysis treatment, a patient is said to achieve dry weight if the postdialysis weight is within 0.5 kg of the prescribed dry weight. If the patient achieved dry weight for at least 80% of the sessions in a 30-day period, then the patient is said to achieve dry weight for that interval.

**Table S5: Subgroup Analyses of the Association of Antihypertensive Medication Regimens with All-Cause Mortality in the USRDS and the DCI Cohorts**

|  | **USRDS (N=33,005)^1^** | | **DCI (N=11,291)^2^** | |
| --- | --- | --- | --- | --- |
| **Antihypertensive Regimens** | **HR (95% CI)** | **p** | **HR (95% CI)** | **p** |
| **Overall** |  | |  |  |
| Any β-blocker Regimen Without RAS (BB) | Reference |  | Reference |  |
| Any RAS Regimen Without β-blocker (RAS) | 0.90 (0.82, 0.97) | 0.006 | 0.87 (0.76, 0.98) | 0.021 |
| β-blocker + RAS Combination (BB+RAS) | 0.83 (0.77, 0.89) | < 0.001 | 0.92 (0.82, 1.02) | 0.085 |
| Other Medications and Combinations (OTHER) | 0.95 (0.87, 1.03) | 0.171 | 0.98 (0.86, 1.11) | 0.72 |
| Antihypertensives Discontinued during Follow-up (DC) | 1.88 (1.77, 1.99) | < 0.001 | 1.28 (1.05, 1.55) | 0.012 |
|  |  |  |  |  |
| **Age <65 Years** |  |  |  |  |
| Any β-blocker Regimen Without RAS (BB) | Reference |  | Reference |  |
| Any RAS Regimen Without β-blocker (RAS) | 0.90 (0.78, 1.03) | 0.13 | 0.78 (0.61, 0.99) | 0.05 |
| β-blocker + RAS Combination (BB+RAS) | 0.79 (0.70, 0.90) | 0.00 | 1.04 (0.88, 1.24) | 0.64 |
| Other Medications and Combinations (OTHER) | 1.04 (0.90, 1.21) | 0.60 | 0.86 (0.68, 1.10) | 0.23 |
| Antihypertensives Discontinued during Follow-up (DC) | 1.49 (1.33, 1.66) | <0.001 | 1.23 (0.86, 1.76) | 0.25 |
|  |  |  |  |  |
| **Age ≥ 65 Years** |  |  |  |  |
| Any β-blocker Regimen Without RAS (BB) | Reference |  | Reference |  |
| Any RAS Regimen Without β-blocker (RAS) | 0.89 (0.81, 0.98) | 0.01 | 0.9 (0.77, 1.04) | 0.16 |
| β-blocker + RAS Combination (BB+RAS) | 0.84 (0.77, 0.91) | <0.001 | 0.83 (0.74, 0.94) | 0.00 |
| Other Medications and Combinations (OTHER) | 0.90 (0.82, 0.99) | 0.03 | 0.98 (0.85, 1.14) | 0.82 |
| Antihypertensives Discontinued during Follow-up (DC) | 2.03 (1.90, 2.17) | <0.001 | 1.07 (0.86, 1.33) | 0.56 |
|  |  |  |  |  |
| **Whites** |  |  |  |  |
| Any β-blocker Regimen Without RAS (BB) | Reference |  | Reference |  |
| Any RAS Regimen Without β-blocker (RAS) | 0.94 (0.85, 1.05) | 0.27 | 0.92 (0.79, 1.08) | 0.31 |
| β-blocker + RAS Combination (BB+RAS) | 0.79 (0.72, 0.86) | <0.001 | 0.87 (0.77, 0.99) | 0.04 |
| Other Medications and Combinations (OTHER) | 0.96 (0.86, 1.07) | 0.45 | 0.97 (0.82, 1.14) | 0.67 |
| Antihypertensives Discontinued during Follow-up (DC) | 1.88 (1.74, 2.02) | <0.001 | 1.22 (0.97, 1.54) | 0.09 |
|  |  |  |  |  |
| **Blacks** |  |  |  |  |
| Any β-blocker Regimen Without RAS (BB) | Reference |  | Reference |  |
| Any RAS Regimen Without β-blocker (RAS) | 0.88 (0.75, 1.02) | 0.09 | 0.82 (0.65, 1.04) | 0.10 |
| β-blocker + RAS Combination (BB+RAS) | 0.94 (0.82, 1.07) | 0.36 | 1.00 (0.84, 1.20) | 0.99 |
| Other Medications and Combinations (OTHER) | 0.93 (0.80, 1.07) | 0.32 | 1.01 (0.80, 1.28) | 0.92 |
| Antihypertensives Discontinued during Follow-up (DC) | 1.93 (1.72, 2.16) | <0.001 | 1.19 (0.83, 1.72) | 0.35 |
|  |  |  |  |  |
| **Hispanics** |  |  |  |  |
| Any β-blocker Regimen Without RAS (BB) | Reference |  | Reference |  |
| Any RAS Regimen Without β-blocker (RAS) | 0.76 (0.61, 0.94) | 0.01 | *** |  |
| β-blocker + RAS Combination (BB+RAS) | 0.76 (0.62, 0.93) | 0.01 | *** |  |
| Other Medications and Combinations (OTHER) | 0.92 (0.72, 1.18) | 0.51 | *** |  |
| Antihypertensives Discontinued during Follow-up (DC) | 1.80 (1.51, 2.15) | <0.001 | *** |  |
|  |  |  |  |  |
| **CVD=YES** |  |  |  |  |
| Any β-blocker Regimen Without RAS (BB) | Reference |  | Reference |  |
| Any RAS Regimen Without β-blocker (RAS) | 0.88 (0.81, 0.96) | 0.01 | 0.85 (0.74, 0.99) | 0.04 |
| β-blocker + RAS Combination (BB+RAS) | 0.80 (0.74, 0.86) | <0.001 | 0.88 (0.78, 0.99) | 0.03 |
| Other Medications and Combinations (OTHER) | 0.91 (0.83, 1.00) | 0.04 | 1.05 (0.90, 1.22) | 0.57 |
| Antihypertensives Discontinued during Follow-up (DC) | 1.89 (1.77, 2.01) | <.0001 | 1.17 (0.94, 1.45) | 0.16 |
|  |  |  |  |  |
| **CVD=NO** |  |  |  |  |
| Any β-blocker Regimen Without RAS (BB) | Reference |  | Reference |  |
| Any RAS Regimen Without β-blocker (RAS) | 0.88 (0.75, 1.04) | 0.13 | 0.91 (0.74, 1.12) | 0.38 |
| β-blocker + RAS Combination (BB+RAS) | 0.90 (0.78, 1.04) | 0.15 | 0.95 (0.79, 1.14) | 0.58 |
| Other Medications and Combinations (OTHER) | 1.02 (0.88, 1.19) | 0.78 | 0.88 (0.70, 1.10) | 0.26 |
| Antihypertensives Discontinued during Follow-up (DC) | 1.84 (1.63, 2.08) | <0.001 | 1.43 (1.05, 1.96) | 0.02 |
|  |  |  |  |  |
| **CHF=YES** |  |  |  |  |
| Any β-blocker Regimen Without RAS (BB) | Reference |  | Reference |  |
| Any RAS Regimen Without β-blocker (RAS) | 0.85 (0.78, 0.94) | 0.00 | 0.85 (0.71, 1.01) | 0.06 |
| β-blocker + RAS Combination (BB+RAS) | 0.83 (0.76, 0.90) | <.0001 | 0.93 (0.82, 1.06) | 0.29 |
| Other Medications and Combinations (OTHER) | 0.90 (0.81, 0.99) | 0.03 | 1.08 (0.92, 1.27) | 0.32 |
| Antihypertensives Discontinued during Follow-up (DC) | 1.92 (1.80, 2.05) | <.0001 | 1.11 (0.86, 1.43) | 0.44 |
|  |  |  |  |  |
| **CHF=NO** |  |  |  |  |
| Any β-blocker Regimen Without RAS (BB) | Reference |  | Reference |  |
| Any RAS Regimen Without β-blocker (RAS) | 0.99 (0.88, 1.12) | 0.90 | 0.89 (0.73, 1.08) | 0.23 |
| β-blocker + RAS Combination (BB+RAS) | 0.81 (0.72, 0.92) | 0.00 | 0.85 (0.74, 0.99) | 0.04 |
| Other Medications and Combinations (OTHER) | 0.99 (0.87, 1.12) | 0.85 | 0.89 (0.73, 1.09) | 0.25 |
| Antihypertensives Discontinued during Follow-up (DC) | 1.87 (1.69, 2.07) | <0.001 | 1.35 (1.04, 1.75) | 0.02 |
|  |  |  |  |  |
| **DM=YES** |  |  |  |  |
| Any β-blocker Regimen Without RAS (BB) | Reference |  | Reference |  |
| Any RAS Regimen Without β-blocker (RAS) | 0.87 (0.80, 0.95) | 0.00 | 0.88 (0.75, 1.03) | 0.11 |
| β-blocker + RAS Combination (BB+RAS) | 0.84 (0.77, 0.90) | <0.001 | 0.94 (0.84, 1.05) | 0.28 |
| Other Medications and Combinations (OTHER) | 0.95 (0.86, 1.04) | 0.25 | 0.99 (0.84, 1.16) | 0.88 |
| Antihypertensives Discontinued during Follow-up (DC) | 1.88 (1.76, 2.01) | <0.001 | 1.33 (1.06, 1.65) | 0.01 |
|  |  |  |  |  |
| **DM=NO** |  |  |  |  |
| Any β-blocker Regimen Without RAS (BB) | Reference |  | Reference |  |
| Any RAS Regimen Without β-blocker (RAS) | 0.98 (0.84, 1.14) | 0.80 | 0.78 (0.62, 0.99) | 0.04 |
| β-blocker + RAS Combination (BB+RAS) | 0.80 (0.69, 0.92) | 0.00 | 0.83 (0.70, 1.00) | 0.05 |
| Other Medications and Combinations (OTHER) | 0.95 (0.81, 1.10) | 0.48 | 0.87 (0.71, 1.08) | 0.21 |
| Antihypertensives Discontinued during Follow-up (DC) | 1.88 (1.69, 2.10) | <0.001 | 1.12 (0.81, 1.53) | 0.50 |

Abbreviations: USRDS, United States Renal Data System; DCI, Dialysis Clinic, Inc.; HR, Hazard Ratio; CI, Confidence Interval; RAS, renin-angiotensin system blocking drugs

*** Too few patients to compute.

**^1^ USRDS Cohort:** HR from discrete time marginal structural models adjusting for the following:

Baseline factors: age, sex, race-ethnicity, insurance status, body mass index, cause of end stage renal disease, albumin, and hemoglobin

Baseline and time-varying factors: comorbidities (diabetes, cardiovascular disease, congestive heart failure, chronic obstructive pulmonary disease, comorbidity index) and antihypertensive regimen in the prior month

**^2^ DCI Cohort:** HR from discrete time marginal structural models adjusting for the following:

Baseline factors: age, sex, race-ethnicity, insurance status, and cause of end stage renal disease

Baseline and time-varying factors: comorbidities (diabetes, cardiovascular disease, congestive heart failure, chronic obstructive pulmonary disease, comorbidity index), cardiovascular hospitalization, end-stage renal disease related factors (body mass index, systolic blood pressure, systolic blood pressure variability, relative volume removed, dry weight attainment, adherence), laboratory data (albumin, , calcium-phosphorus product, hemoglobin, ferritin and Kt/V_UREA_) and antihypertensive regimen in the prior month

**Table S6: Subgroup Analyses of the Association of Antihypertensive Medication Regimens with Cardiovascular Mortality in the USRDS and the DCI Cohorts**

|  | **USRDS (N=33,005)** | | **DCI (N=11,291)** | |
| --- | --- | --- | --- | --- |
| **Antihypertensive Regimens** | **HR (95% CI) ^1^** | **p** | **HR (95% CI) ^1^** | **p** |
| **Overall** |  | |  |  |
| Any β-blocker Regimen Without RAS (BB) | Reference |  | Reference |  |
| Any RAS Regimen Without β-blocker (RAS) | 0.84 (0.75, 0.95) | 0.003 | 0.88 (0.71, 1.07) | 0.181 |
| β-blocker + RAS Combination (BB+RAS) | 0.84 (0.75, 0.93) | <0.001 | 0.96 (0.83, 1.09) | 0.49 |
| Other Medications and Combinations (OTHER) | 0.86 (0.76, 0.98) | 0.016 | 0.97 (0.80, 1.17) | 0.71 |
| Antihypertensives Discontinued during Follow-up (DC) | 1.65 (1.52, 1.80) | <0.001 | 1.18 (0.89, 1.57) | 0.24 |
|  |  |  |  |  |
| **Age <65 Years** |  |  |  |  |
| Any β-blocker Regimen Without RAS (BB) | Reference |  | Reference |  |
| Any RAS Regimen Without β-blocker (RAS) | 0.81 (0.66, 1.00) | 0.05 | 0.86 (0.6, 1.23) | 0.41 |
| β-blocker + RAS Combination (BB+RAS) | 0.83 (0.69, 0.99) | 0.04 | 1.18 (0.96, 1.45) | 0.109 |
| Other Medications and Combinations (OTHER) | 0.97 (0.77, 1.21) | 0.77 | 0.96 (0.68, 1.36) | 0.84 |
| Antihypertensives Discontinued during Follow-up (DC) | 1.30 (1.11, 1.53) | 0.00 | 1.25 (0.77, 2.03) | 0.37 |
|  |  |  |  |  |
| **Age ≥ 65 Years** |  |  |  |  |
| Any β-blocker Regimen Without RAS (BB) | Reference |  | Reference |  |
| Any RAS Regimen Without β-blocker (RAS) | 0.85 (0.74, 0.97) | 0.02 | 0.85 (0.67, 1.06) | 0.153 |
| β-blocker + RAS Combination (BB+RAS) | 0.84 (0.75, 0.94) | 0.00 | 0.81 (0.68, 0.98) | 0.028 |
| Other Medications and Combinations (OTHER) | 0.83 (0.72, 0.95) | 0.01 | 0.91 (0.74, 1.13) | 0.42 |
| Antihypertensives Discontinued during Follow-up (DC) | 1.80 (1.63, 1.97) | <0.001 | 0.93 (0.66, 1.30) | 0.66 |
|  |  |  |  |  |
| **Whites** |  |  |  |  |
| Any β-blocker Regimen Without RAS (BB) | Reference |  | Reference |  |
| Any RAS Regimen Without β-blocker (RAS) | 0.81 (0.70, 0.95) | 0.01 | 0.88 (0.69, 1.12) | 0.30 |
| β-blocker + RAS Combination (BB+RAS) | 0.76 (0.67, 0.87) | <0.001 | 0.88 (0.73, 1.05) | 0.142 |
| Other Medications and Combinations (OTHER) | 0.83 (0.70, 0.97) | 0.02 | 0.93 (0.74, 1.18) | 0.57 |
| Antihypertensives Discontinued during Follow-up (DC) | 1.66 (1.49, 1.85) | <0.001 | 0.95 (0.67, 1.37) | 0.80 |
|  |  |  |  |  |
| **Blacks** |  |  |  |  |
| Any β-blocker Regimen Without RAS (BB) | Reference |  | Reference |  |
| Any RAS Regimen Without β-blocker (RAS) | 0.99 (0.80, 1.22) | 0.91 | 0.93 (0.65, 1.34) | 0.69 |
| β-blocker + RAS Combination (BB+RAS) | 0.97 (0.81, 1.17) | 0.76 | 1.11 (0.87, 1.42) | 0.40 |
| Other Medications and Combinations (OTHER) | 0.98 (0.80, 1.21) | 0.87 | 1.10 (0.78, 1.56) | 0.58 |
| Antihypertensives Discontinued during Follow-up (DC) | 1.61 (1.37, 1.90) | <0.001 | 1.63 (1.03, 2.59) | 0.037 |
|  |  |  |  |  |
| **Hispanics** |  |  |  |  |
| Any β-blocker Regimen Without RAS (BB) | Reference |  | Reference |  |
| Any RAS Regimen Without β-blocker (RAS) | 0.70 (0.51, 0.96) | 0.03 | *** |  |
| β-blocker + RAS Combination (BB+RAS) | 0.84 (0.63, 1.10) | 0.21 | *** |  |
| Other Medications and Combinations (OTHER) | 0.74 (0.52, 1.05) | 0.09 | *** |  |
| Antihypertensives Discontinued during Follow-up (DC) | 1.67 (1.31, 2.13) | <0.001 | *** |  |
|  |  |  |  |  |
| **CVD=YES** |  |  |  |  |
| Any β-blocker Regimen Without RAS (BB) | Reference |  | Reference |  |
| Any RAS Regimen Without β-blocker (RAS) | 0.86 (0.76, 0.97) | 0.02 | 0.89 (0.71, 1.11) | 0.30 |
| β-blocker + RAS Combination (BB+RAS) | 0.81 (0.73, 0.91) | 0.00 | 0.98 (0.84, 1.14) | 0.77 |
| Other Medications and Combinations (OTHER) | 0.82 (0.72, 0.94) | 0.00 | 1.14 (0.92, 1.42) | 0.24 |
| Antihypertensives Discontinued during Follow-up (DC) | 1.66 (1.52, 1.81) | <0.001 | 0.99 (0.72, 1.38) | 0.97 |
|  |  |  |  |  |
| **CVD=NO** |  |  |  |  |
| Any β-blocker Regimen Without RAS (BB) | Reference |  | Reference |  |
| Any RAS Regimen Without β-blocker (RAS) | 0.72 (0.57, 0.92) | 0.01 | 0.89 (0.63, 1.26) | 0.51 |
| β-blocker + RAS Combination (BB+RAS) | 0.92 (0.74, 1.14) | 0.43 | 0.83 (0.63, 1.09) | 0.173 |
| Other Medications and Combinations (OTHER) | 1.01 (0.80, 1.27) | 0.95 | 0.69 (0.46, 1.03) | 0.073 |
| Antihypertensives Discontinued during Follow-up (DC) | 1.73 (1.43, 2.09) | <0.001 | 1.50 (0.93, 2.42) | 0.100 |
|  |  |  |  |  |
| **CHF=YES** |  |  |  |  |
| Any β-blocker Regimen Without RAS (BB) | Reference |  | Reference |  |
| Any RAS Regimen Without β-blocker (RAS) | 0.87 (0.76, 0.99) | 0.04 | 0.81 (0.63, 1.04) | 0.091 |
| β-blocker + RAS Combination (BB+RAS) | 0.87 (0.78, 0.97) | 0.01 | 0.99 (0.85, 1.17) | 0.94 |
| Other Medications and Combinations (OTHER) | 0.85 (0.74, 0.99) | 0.03 | 0.97 (0.76, 1.23) | 0.79 |
| Antihypertensives Discontinued during Follow-up (DC) | 1.74 (1.58, 1.91) | <0.001 | 0.95 (0.65, 1.38) | 0.78 |
|  |  |  |  |  |
| **CHF=NO** |  |  |  |  |
| Any β-blocker Regimen Without RAS (BB) | Reference |  | Reference |  |
| Any RAS Regimen Without β-blocker (RAS) | 0.81 (0.66, 0.99) | 0.04 | 0.94 (0.69, 1.30) | 0.73 |
| β-blocker + RAS Combination (BB+RAS) | 0.76 (0.63, 0.91) | 0.00 | 0.81 (0.64, 1.01) | 0.060 |
| Other Medications and Combinations (OTHER) | 0.84 (0.69, 1.03) | 0.09 | 0.96 (0.71, 1.30) | 0.80 |
| Antihypertensives Discontinued during Follow-up (DC) | 1.58 (1.35, 1.85) | <0.001 | 1.35 (0.90, 2.02) | 0.147 |
|  |  |  |  |  |
| **DM=YES** |  |  |  |  |
| Any β-blocker Regimen Without RAS (BB) | Reference |  | Reference |  |
| Any RAS Regimen Without β-blocker (RAS) | 0.82 (0.72, 0.93) | 0.00 | 0.89 (0.70, 1.12) | 0.33 |
| β-blocker + RAS Combination (BB+RAS) | 0.86 (0.77, 0.96) | 0.01 | 0.99 (0.86, 1.14) | 0.85 |
| Other Medications and Combinations (OTHER) | 0.86 (0.75, 0.98) | 0.03 | 0.94 (0.74, 1.20) | 0.65 |
| Antihypertensives Discontinued during Follow-up (DC) | 1.68 (1.53, 1.84) | <0.001 | 1.22 (0.87, 1.71) | 0.25 |
|  |  |  |  |  |
| **DM=NO** |  |  |  |  |
| Any β-blocker Regimen Without RAS (BB) | Reference |  | Reference |  |
| Any RAS Regimen Without β-blocker (RAS) | 0.92 (0.73, 1.16) | 0.49 | 0.74 (0.53, 1.03) | 0.073 |
| β-blocker + RAS Combination (BB+RAS) | 0.79 (0.64, 0.96) | 0.02 | 0.84 (0.64, 1.10) | 0.21 |
| Other Medications and Combinations (OTHER) | 0.89 (0.71, 1.13) | 0.35 | 0.87 (0.63, 1.21) | 0.41 |
| Antihypertensives Discontinued during Follow-up (DC) | 1.59 (1.34, 1.88) | <0.001 | 0.95 (0.59, 1.51) | 0.82 |

*** Too few patients to compute.

Abbreviations: USRDS, United States Renal Data System; DCI, Dialysis Clinic, Inc.; HR, Hazard Ratio; CI, Confidence Interval; RAS, renin-angiotensin system blocking drugs

*** Too few patients to compute.

**^1^ USRDS Cohort:** HR from discrete time marginal structural models adjusting for the following:

Baseline factors: age, sex, race-ethnicity, insurance status, body mass index, cause of end stage renal disease, albumin, and hemoglobin

Baseline and time-varying factors: comorbidities (diabetes, cardiovascular disease, congestive heart failure, chronic obstructive pulmonary disease, comorbidity index) and antihypertensive regimen in the prior month

**^2^ DCI Cohort:** HR from discrete time marginal structural models adjusting for the following:

Baseline factors: age, sex, race-ethnicity, insurance status, and cause of end stage renal disease

Baseline and time-varying factors: comorbidities (diabetes, cardiovascular disease, congestive heart failure, chronic obstructive pulmonary disease, comorbidity index), cardiovascular hospitalization, end-stage renal disease related factors (body mass index, systolic blood pressure, systolic blood pressure variability, relative volume removed, dry weight attainment, adherence), laboratory data (albumin, , calcium-phosphorus product, hemoglobin, ferritin and Kt/V_UREA_) and antihypertensive regimen in the prior month

**Table S7: Association of Antihypertensive Medication Classes with Cardiovascular Hospitalizations or Any-Cause Death among 7,848 Incident Hemodialysis Patients of the DCI-Medicare Subcohort**

| **Antihypertensive Regimens** | **HR (95% CI) ^1^** | **p** |
| --- | --- | --- |
| **Overall** |  | |
| Any β-blocker Regimen Without RAS (BB) | Ref |  |
| Any RAS Regimen Without β-blocker (RAS) | 0.96 (0.91, 1.02) | 0.18 |
| β-blocker + RAS Combination (BB+RAS) | 1.02 (0.97, 1.07) | 0.46 |
| Other Medications and Combinations (OTHER) | 1.02 (0.95, 1.09) | 0.54 |
| Antihypertensives Discontinued during Follow-up (DC) | 0.96 (0.87, 1.06) | 0.41 |
|  |  |  |
| **Age <65 Years** |  |  |
| Any β-blocker Regimen Without RAS (BB) | Ref |  |
| Any RAS Regimen Without β-blocker (RAS) | 0.92 (0.84, 1.01) | 0.07 |
| β-blocker + RAS Combination (BB+RAS) | 1.03 (0.96, 1.11) | 0.45 |
| Other Medications and Combinations (OTHER) | 0.97 (0.87, 1.08) | 0.58 |
| Antihypertensives Discontinued during Follow-up (DC) | 0.93 (0.79, 1.10) | 0.39 |
|  |  |  |
| **Age ≥ 65 Years** |  |  |
| Any β-blocker Regimen Without RAS (BB) | Ref |  |
| Any RAS Regimen Without β-blocker (RAS) | 0.96 (0.89, 1.04) | 0.33 |
| β-blocker + RAS Combination (BB+RAS) | 0.98 (0.92, 1.04) | 0.47 |
| Other Medications and Combinations (OTHER) | 1.04 (0.96, 1.13) | 0.36 |
| Antihypertensives Discontinued during Follow-up (DC) | 0.99 (0.88, 1.12) | 0.86 |
|  |  |  |
| **Whites** |  |  |
| Any β-blocker Regimen Without RAS (BB) | Ref |  |
| Any RAS Regimen Without β-blocker (RAS) | 0.98 (0.91, 1.07) | 0.70 |
| β-blocker + RAS Combination (BB+RAS) | 1.00 (0.94, 1.07) | 0.89 |
| Other Medications and Combinations (OTHER) | 0.99 (0.91, 1.08) | 0.83 |
| Antihypertensives Discontinued during Follow-up (DC) | 0.96 (0.85, 1.08) | 0.50 |
|  |  |  |
| **Blacks** |  |  |
| Any β-blocker Regimen Without RAS (BB) | Ref |  |
| Any RAS Regimen Without β-blocker (RAS) | 0.90 (0.81, 0.99) | 0.03 |
| β-blocker + RAS Combination (BB+RAS) | 0.97 (0.89, 1.05) | 0.47 |
| Other Medications and Combinations (OTHER) | 1.03 (0.92, 1.15) | 0.64 |
| Antihypertensives Discontinued during Follow-up (DC) | 1.05 (0.89, 1.23) | 0.58 |
|  |  |  |
| **CVD=YES** |  |  |
| Any β-blocker Regimen Without RAS (BB) | Ref |  |
| Any RAS Regimen Without β-blocker (RAS) | 0.93 (0.87, 1.01) | 0.07 |
| β-blocker + RAS Combination (BB+RAS) | 0.98 (0.92, 1.04) | 0.46 |
| Other Medications and Combinations (OTHER) | 1.02 (0.94, 1.11) | 0.65 |
| Antihypertensives Discontinued during Follow-up (DC) | 0.96 (0.85, 1.08) | 0.50 |
|  |  |  |
| **CVD=NO** |  |  |
| Any β-blocker Regimen Without RAS (BB) | Ref |  |
| Any RAS Regimen Without β-blocker (RAS) | 1.01 (0.92, 1.12) | 0.82 |
| β-blocker + RAS Combination (BB+RAS) | 1.06 (0.98, 1.16) | 0.15 |
| Other Medications and Combinations (OTHER) | 1.04 (0.94, 1.16) | 0.44 |
| Antihypertensives Discontinued during Follow-up (DC) | 1.04 (0.88, 1.23) | 0.66 |
|  |  |  |
| **CHF=YES** |  |  |
| Any β-blocker Regimen Without RAS (BB) | Ref |  |
| Any RAS Regimen Without β-blocker (RAS) | 0.98 (0.90, 1.07) | 0.66 |
| β-blocker + RAS Combination (BB+RAS) | 1.01 (0.94, 1.07) | 0.86 |
| Other Medications and Combinations (OTHER) | 1.09 (1.00, 1.19) | 0.06 |
| Antihypertensives Discontinued during Follow-up (DC) | 1.00 (0.89, 1.14) | 0.94 |
|  |  |  |
| **CHF=NO** |  |  |
| Any β-blocker Regimen Without RAS (BB) | Ref |  |
| Any RAS Regimen Without β-blocker (RAS) | 0.93 (0.85, 1.01) | 0.08 |
| β-blocker + RAS Combination (BB+RAS) | 0.98 (0.91, 1.05) | 0.55 |
| Other Medications and Combinations (OTHER) | 0.96 (0.87, 1.05) | 0.36 |
| Antihypertensives Discontinued during Follow-up (DC) | 0.92 (0.78, 1.07) | 0.27 |
|  |  |  |
| **DM=YES** |  |  |
| Any β-blocker Regimen Without RAS (BB) | Ref |  |
| Any RAS Regimen Without β-blocker (RAS) | 0.92 (0.86, 0.99) | 0.03 |
| β-blocker + RAS Combination (BB+RAS) | 1.01 (0.95, 1.07) | 0.73 |
| Other Medications and Combinations (OTHER) | 1.06 (0.98, 1.15) | 0.13 |
| Antihypertensives Discontinued during Follow-up (DC) | 1.00 (0.89, 1.13) | 0.96 |
|  |  |  |
| **DM=NO** |  |  |
| Any β-blocker Regimen Without RAS (BB) | Ref |  |
| Any RAS Regimen Without β-blocker (RAS) | 0.99 (0.90, 1.10) | 0.89 |
| β-blocker + RAS Combination (BB+RAS) | 1.00 (0.92, 1.10) | 0.93 |
| Other Medications and Combinations (OTHER) | 0.91 (0.81, 1.02) | 0.11 |
| Antihypertensives Discontinued during Follow-up (DC) | 0.95 (0.80, 1.13) | 0.60 |

Abbreviations: DCI, Dialysis Clinic, Inc.; HR, Hazard Ratio; CI, Confidence Interval; BB, β-blocker; RAS, renin-angiotensin system blocking drugs

**^1^** HR from discrete time marginal structural models adjusting for the following:

Baseline factors: age, sex, race-ethnicity, insurance status, and cause of end stage renal disease

Baseline and time-varying factors: comorbidities (diabetes, cardiovascular disease, congestive heart failure, chronic obstructive pulmonary disease, comorbidity index), end-stage renal disease related factors (body mass index, systolic blood pressure, systolic blood pressure variability, relative volume removed, dry weight attainment, adherence), laboratory data (albumin, calcium-phosphorus product, hemoglobin, ferritin and Kt/V_UREA_) and antihypertensive regimen in the prior month.
